# Crystal structure of 2-(4-methyl­piperazin-1-yl)quinoline-3-carbaldehyde

**DOI:** 10.1107/S2056989015020186

**Published:** 2015-10-31

**Authors:** R Nivedita Desai, S Sreenivasa, S. Naveen, N. K. Lokanath, P. A. Suchetan, D. B. Aruna Kumar

**Affiliations:** a Department of Chemistry, University College of Science, Tumkur University, Tumkur 572 103, India; b Institution of Excellence, University of Mysore, Mysuru-6, India; c Department of Physics, University of Mysore, Mysuru-6, India

**Keywords:** crystal structure, quinolines, piperazines

## Abstract

In the title compound, C_15_H_17_N_3_O, the aldehyde group is twisted relative to the quinoline group by17.6 (2)° due to the presence of a bulky piperazinyl group in the *ortho* position. The piperazine N atom attached to the aromatic ring is *sp* ^3^-hybridized and the dihedral angle between the mean planes through the the six piperazine ring atoms and through the quinoline ring system is 40.59 (7)°. Both piperazine substituents are in equatorial positions.

## Related literature   

For biological activity of quinoline derivatives, see: Nasveld *et al.* (2005 ▸); Eswaran *et al.* (2009 ▸); Leatham *et al.* (1983 ▸); Muruganantham *et al.* (2004 ▸); Maguire *et al.* (1994 ▸); Wilson *et al.* (1992 ▸); Strekowski *et al.* (1991 ▸). For photonic and electronic properties of poly-substituted quinolines, see: Gyoten *et al.* (2003 ▸).
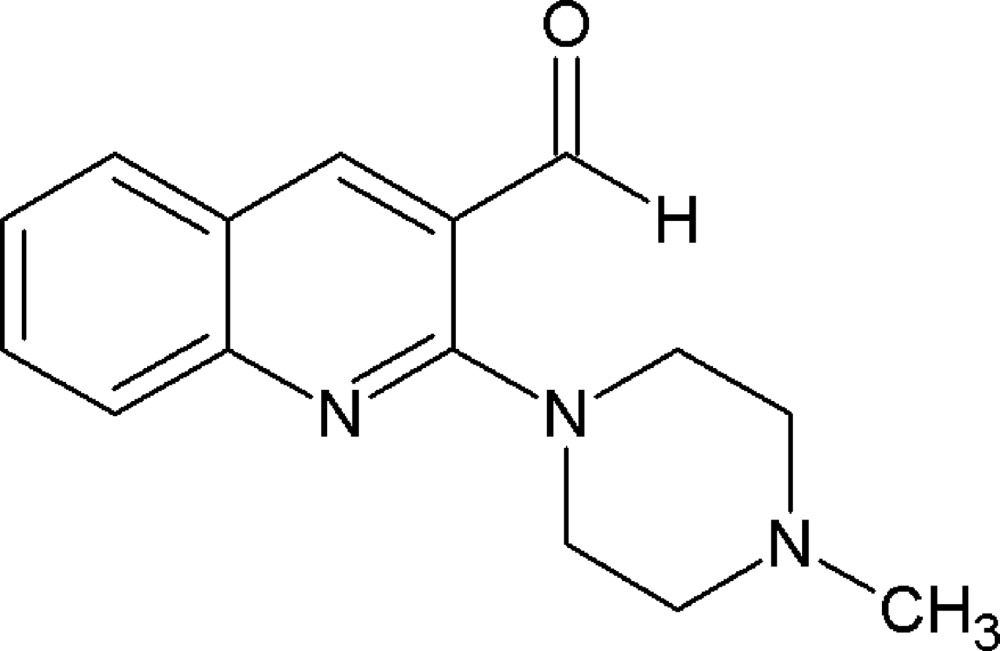


## Experimental   

### Crystal data  

C_15_H_17_N_3_O
*M* *_r_* = 255.32
Monoclinic, 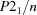

*a* = 12.3282 (4) Å
*b* = 5.8935 (2) Å
*c* = 18.9202 (7) Å
β = 103.591 (2)°
*V* = 1336.18 (8) Å^3^
*Z* = 4
Cu *K*α radiation
μ = 0.65 mm^−1^
*T* = 296 K
0.28 × 0.26 × 0.24 mm

### Data collection  

Bruker APEXII CCD diffractometer
Absorption correction: multi-scan (*SADABS*; Bruker, 2009 ▸) *T*_min_ = 0.838, *T*_max_ = 0.859
9762 measured reflections
2181 independent reflections
1859 reflections with *I* > 2σ(*I*)
*R*_int_ = 0.048

### Refinement  

*R*[*F* ^2^ > 2σ(*F* ^2^)] = 0.054
*wR*(*F* ^2^) = 0.162
*S* = 1.06
2181 reflections
173 parameters
H-atom parameters constrained
Δρ_max_ = 0.22 e Å^−3^
Δρ_min_ = −0.24 e Å^−3^

### 

Data collection: *APEX2* (Bruker, 2009 ▸); cell refinement: *APEX2* and *SAINT-Plus* (Bruker, 2009 ▸); data reduction: *SAINT-Plus* and *XPREP* (Bruker, 2009 ▸); program(s) used to solve structure: *SHELXS97* (Sheldrick, 2008 ▸); program(s) used to refine structure: *SHELXL97* (Sheldrick, 2008 ▸); molecular graphics: *Mercury* (Macrae *et al.*, 2008 ▸); software used to prepare material for publication: *SHELXL97*.

## Supplementary Material

Crystal structure: contains datablock(s) I. DOI: 10.1107/S2056989015020186/gk2647sup1.cif

Structure factors: contains datablock(s) I. DOI: 10.1107/S2056989015020186/gk2647Isup2.hkl

Supporting information file. DOI: 10.1107/S2056989015020186/gk2647Isup3.cml

**Fig. 1.**
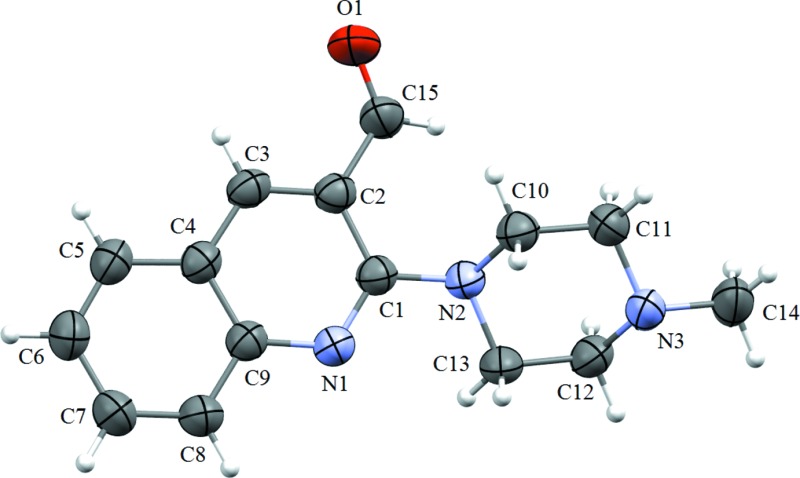
Molecular structure of the title compound with displacement ellipsoids drawn at the 50% probability level.

. DOI: 10.1107/S2056989015020186/gk2647fig1.tif
Mol­ecular structure of the title compound with displacement ellipsoids drawn at the 50% probability level.

CCDC reference: 1433198

Additional supporting information:  crystallographic information; 3D view; checkCIF report

## supplementary crystallographic information

### S1. Introduction

Quinoline and its derivatives have been well known in pharmaceutical chemistry because of their wide spectrum of biological activities and their presence in naturally occurring compounds. They have been shown to possess anti­malarial (Nasveld *et al.*, 2005), anti­biotic (Eswaran *et al.*, 2009), anti­cancer (Denny *et al.*, 1983), anti-inflammatory (Muruganantham *et al*., 2004), anti­hypertensive (Maguire *et al.*, 1994), tyrokinase PDGF-RTK inhibition (Wilson *et al.*, 1992) and anti-HIV properties (Strekowski *et al.*, 1991). In addition, polysubstituted quinoline can achieve hierchical self-assembly into variety of meso and nano structures with enhanced photonic and electronic properties (Gyoten *et al.*, 2003). In this view the title compound was synthesized to study its crystal structure.

### S2.1. Synthesis and crystallization

2-Chloro­quinoline-3-carbaldehyde (0.42 g, 0.00351 mmol), *N*-methyl piperazine (0.14 g, 0.00351 mmol) and anhydrous K_2_CO_3_ (1.0 g, 0.002920 mmol) were refluxed for 24 hrs in DMF. The progress of the reaction was monitored by thin layer chromatography. After the completion of the reaction, the reaction mixture was poured into water and extracted to ethyl acetate. The organic layer was washed with water, dried and concentrated under vacuum using rotary evaporator. Single crystals of the title compound were obtained by slow evaporation of the ethyl acetate solution at room temperature (27^o^C).

### S2.2. Refinement

Crystal data, data collection and structure refinement details are summarized in Table 1. All H atoms were positioned with idealized geometry using a riding model with C—H = 0.93-0.97 Å. All H atoms were refined with isotropic displacement parameters (set to 1.2 times of the Ueq of the parent C atom).

### S3. Results and discussion

The crystal packing of the compound does not feature any specific strong or weak inter­molecular inter­actions.

### Crystal data

**Table:**

C_15_H_17_N_3_O	Prism
*M**_r_* = 255.32	*D*_x_ = 1.269 Mg m^−^^3^
Monoclinic, *P*2_1_/*n*	Melting point: 384 K
Hall symbol: -P 2yn	Cu *K*α radiation, λ = 1.54178 Å
*a* = 12.3282 (4) Å	Cell parameters from 143 reflections
*b* = 5.8935 (2) Å	θ = 3.9–64.5°
*c* = 18.9202 (7) Å	µ = 0.65 mm^−^^1^
β = 103.591 (2)°	*T* = 296 K
*V* = 1336.18 (8) Å^3^	Prism, colourless
*Z* = 4	0.28 × 0.26 × 0.24 mm
*F*(000) = 544	

### Data collection

**Table:**

Bruker APEXII CCD diffractometer	2181 independent reflections
Radiation source: fine-focus sealed tube	1859 reflections with *I* > 2σ(*I*)
Graphite monochromator	*R*_int_ = 0.048
phi and φ scans	θ_max_ = 64.5°, θ_min_ = 3.9°
Absorption correction: multi-scan (*SADABS*; Bruker, 2009)	*h* = −14→14
*T*_min_ = 0.838, *T*_max_ = 0.859	*k* = −6→6
9762 measured reflections	*l* = −21→21

### Refinement

**Table:**

Refinement on *F*^2^	Primary atom site location: structure-invariant direct methods
Least-squares matrix: full	Secondary atom site location: difference Fourier map
*R*[*F*^2^ > 2σ(*F*^2^)] = 0.054	Hydrogen site location: inferred from neighbouring sites
*wR*(*F*^2^) = 0.162	H-atom parameters constrained
*S* = 1.06	*w* = 1/[σ^2^(*F*_o_^2^) + (0.1151*P*)^2^ + 0.0654*P*] where *P* = (*F*_o_^2^ + 2*F*_c_^2^)/3
2181 reflections	(Δ/σ)_max_ < 0.001
173 parameters	Δρ_max_ = 0.22 e Å^−^^3^
0 restraints	Δρ_min_ = −0.24 e Å^−^^3^

### Special details

**Table:**

Geometry. All s.u.'s (except the s.u. in the dihedral angle between two l.s. planes) are estimated using the full covariance matrix. The cell s.u.'s are taken into account individually in the estimation of s.u.'s in distances, angles and torsion angles; correlations between s.u.'s in cell parameters are only used when they are defined by crystal symmetry. An approximate (isotropic) treatment of cell s.u.'s is used for estimating s.u.'s involving l.s. planes.
Refinement. Refinement of *F*^2^ against ALL reflections. The weighted *R*-factor *wR* and goodness of fit *S* are based on *F*^2^, conventional *R*-factors *R* are based on *F*, with *F* set to zero for negative *F*^2^. The threshold expression of *F*^2^ > 2σ(*F*^2^) is used only for calculating *R*-factors(gt) *etc*. and is not relevant to the choice of reflections for refinement. *R*-factors based on *F*^2^ are statistically about twice as large as those based on *F*, and *R*- factors based on ALL data will be even larger.

### Fractional atomic coordinates and isotropic or equivalent isotropic displacement parameters (Å^2^)

**Table:**

	*x*	*y*	*z*	*U*_iso_*/*U*_eq_	
N1	0.44347 (10)	0.7887 (2)	0.60405 (6)	0.0446 (4)	
N2	0.31637 (10)	0.4894 (2)	0.58318 (6)	0.0443 (4)	
N3	0.15374 (11)	0.2821 (2)	0.46867 (7)	0.0492 (4)	
O1	0.33529 (13)	0.4038 (3)	0.79890 (7)	0.0800 (5)	
C1	0.38623 (12)	0.6362 (3)	0.63074 (7)	0.0415 (4)	
C2	0.39605 (12)	0.6082 (3)	0.70773 (8)	0.0435 (4)	
C3	0.46317 (13)	0.7537 (3)	0.75426 (8)	0.0456 (4)	
H3	0.4690	0.7410	0.8040	0.055*	
C4	0.52382 (12)	0.9229 (3)	0.72789 (8)	0.0429 (4)	
C5	0.59503 (14)	1.0786 (3)	0.77313 (9)	0.0517 (5)	
H5	0.6035	1.0718	0.8232	0.062*	
C6	0.65119 (15)	1.2380 (3)	0.74416 (10)	0.0571 (5)	
H6	0.6980	1.3397	0.7744	0.068*	
C7	0.63874 (16)	1.2497 (3)	0.66853 (10)	0.0580 (5)	
H7	0.6768	1.3606	0.6490	0.070*	
C8	0.57153 (14)	1.1003 (3)	0.62334 (9)	0.0516 (5)	
H8	0.5648	1.1094	0.5734	0.062*	
C9	0.51232 (12)	0.9326 (3)	0.65156 (8)	0.0430 (4)	
C10	0.19665 (13)	0.5037 (3)	0.58101 (8)	0.0477 (4)	
H10A	0.1659	0.6413	0.5560	0.057*	
H10B	0.1861	0.5100	0.6302	0.057*	
C11	0.13648 (13)	0.3007 (3)	0.54226 (9)	0.0517 (5)	
H11A	0.1635	0.1642	0.5694	0.062*	
H11B	0.0573	0.3142	0.5399	0.062*	
C12	0.27299 (14)	0.2697 (3)	0.47221 (9)	0.0522 (5)	
H12A	0.2846	0.2582	0.4234	0.063*	
H12B	0.3038	0.1347	0.4988	0.063*	
C13	0.33266 (14)	0.4768 (3)	0.50926 (8)	0.0516 (5)	
H13A	0.4117	0.4670	0.5106	0.062*	
H13B	0.3031	0.6123	0.4824	0.062*	
C14	0.09682 (18)	0.0831 (4)	0.43236 (9)	0.0660 (6)	
H14A	0.1106	0.0708	0.3846	0.099*	
H14B	0.0181	0.0973	0.4285	0.099*	
H14C	0.1242	−0.0501	0.4600	0.099*	
C15	0.34545 (14)	0.4132 (3)	0.73732 (9)	0.0561 (5)	
H15	0.3201	0.2913	0.7067	0.067*	

### Atomic displacement parameters (Å^2^)

**Table:**

	*U*^11^	*U*^22^	*U*^33^	*U*^12^	*U*^13^	*U*^23^
N1	0.0470 (7)	0.0482 (8)	0.0386 (7)	−0.0039 (6)	0.0102 (6)	0.0002 (5)
N2	0.0423 (7)	0.0538 (9)	0.0381 (7)	−0.0054 (6)	0.0119 (5)	−0.0038 (6)
N3	0.0541 (8)	0.0505 (9)	0.0400 (7)	−0.0114 (6)	0.0047 (6)	0.0021 (6)
O1	0.0885 (10)	0.1024 (13)	0.0487 (8)	−0.0299 (8)	0.0151 (7)	0.0178 (7)
C1	0.0400 (8)	0.0462 (9)	0.0389 (8)	0.0020 (6)	0.0102 (6)	0.0015 (6)
C2	0.0408 (8)	0.0503 (10)	0.0392 (8)	0.0025 (6)	0.0088 (6)	0.0038 (7)
C3	0.0456 (9)	0.0556 (10)	0.0348 (8)	0.0055 (7)	0.0076 (6)	0.0042 (7)
C4	0.0402 (8)	0.0467 (9)	0.0407 (8)	0.0049 (6)	0.0077 (6)	0.0001 (6)
C5	0.0529 (10)	0.0568 (11)	0.0431 (8)	0.0002 (8)	0.0064 (7)	−0.0061 (7)
C6	0.0583 (10)	0.0557 (11)	0.0549 (10)	−0.0102 (8)	0.0086 (8)	−0.0100 (8)
C7	0.0640 (11)	0.0530 (11)	0.0576 (10)	−0.0139 (8)	0.0155 (8)	−0.0007 (8)
C8	0.0574 (10)	0.0544 (11)	0.0439 (8)	−0.0055 (8)	0.0142 (7)	0.0014 (7)
C9	0.0427 (8)	0.0458 (10)	0.0402 (8)	0.0020 (6)	0.0090 (6)	−0.0002 (6)
C10	0.0450 (9)	0.0568 (11)	0.0420 (8)	−0.0011 (7)	0.0117 (6)	0.0002 (7)
C11	0.0481 (9)	0.0603 (11)	0.0465 (9)	−0.0097 (7)	0.0108 (7)	0.0025 (7)
C12	0.0614 (10)	0.0561 (11)	0.0402 (8)	−0.0041 (7)	0.0143 (7)	−0.0042 (7)
C13	0.0513 (9)	0.0644 (11)	0.0424 (8)	−0.0112 (8)	0.0180 (7)	−0.0056 (7)
C14	0.0820 (13)	0.0605 (12)	0.0491 (10)	−0.0239 (9)	0.0026 (9)	0.0008 (8)
C15	0.0567 (10)	0.0634 (12)	0.0449 (9)	−0.0096 (8)	0.0050 (7)	0.0107 (8)

### Geometric parameters (Å, º)

**Table:**

N1—C1	1.315 (2)	C6—H6	0.9300
N1—C9	1.375 (2)	C7—C8	1.364 (3)
N2—C1	1.391 (2)	C7—H7	0.9300
N2—C13	1.4607 (18)	C8—C9	1.406 (2)
N2—C10	1.4693 (19)	C8—H8	0.9300
N3—C14	1.454 (2)	C10—C11	1.505 (2)
N3—C12	1.458 (2)	C10—H10A	0.9700
N3—C11	1.461 (2)	C10—H10B	0.9700
O1—C15	1.202 (2)	C11—H11A	0.9700
C1—C2	1.442 (2)	C11—H11B	0.9700
C2—C3	1.361 (2)	C12—C13	1.510 (2)
C2—C15	1.479 (2)	C12—H12A	0.9700
C3—C4	1.406 (2)	C12—H12B	0.9700
C3—H3	0.9300	C13—H13A	0.9700
C4—C5	1.411 (2)	C13—H13B	0.9700
C4—C9	1.419 (2)	C14—H14A	0.9600
C5—C6	1.357 (3)	C14—H14B	0.9600
C5—H5	0.9300	C14—H14C	0.9600
C6—C7	1.404 (3)	C15—H15	0.9300
			
C1—N1—C9	118.40 (12)	N2—C10—C11	110.18 (13)
C1—N2—C13	116.58 (12)	N2—C10—H10A	109.6
C1—N2—C10	116.60 (12)	C11—C10—H10A	109.6
C13—N2—C10	109.84 (11)	N2—C10—H10B	109.6
C14—N3—C12	110.53 (15)	C11—C10—H10B	109.6
C14—N3—C11	110.40 (13)	H10A—C10—H10B	108.1
C12—N3—C11	109.34 (12)	N3—C11—C10	111.00 (13)
N1—C1—N2	118.89 (12)	N3—C11—H11A	109.4
N1—C1—C2	122.74 (14)	C10—C11—H11A	109.4
N2—C1—C2	118.32 (13)	N3—C11—H11B	109.4
C3—C2—C1	118.36 (14)	C10—C11—H11B	109.4
C3—C2—C15	119.41 (14)	H11A—C11—H11B	108.0
C1—C2—C15	121.90 (15)	N3—C12—C13	110.89 (14)
C2—C3—C4	120.69 (14)	N3—C12—H12A	109.5
C2—C3—H3	119.7	C13—C12—H12A	109.5
C4—C3—H3	119.7	N3—C12—H12B	109.5
C3—C4—C5	123.55 (14)	C13—C12—H12B	109.5
C3—C4—C9	117.08 (14)	H12A—C12—H12B	108.0
C5—C4—C9	119.36 (15)	N2—C13—C12	108.90 (13)
C6—C5—C4	120.59 (15)	N2—C13—H13A	109.9
C6—C5—H5	119.7	C12—C13—H13A	109.9
C4—C5—H5	119.7	N2—C13—H13B	109.9
C5—C6—C7	120.09 (16)	C12—C13—H13B	109.9
C5—C6—H6	120.0	H13A—C13—H13B	108.3
C7—C6—H6	120.0	N3—C14—H14A	109.5
C8—C7—C6	120.80 (16)	N3—C14—H14B	109.5
C8—C7—H7	119.6	H14A—C14—H14B	109.5
C6—C7—H7	119.6	N3—C14—H14C	109.5
C7—C8—C9	120.59 (15)	H14A—C14—H14C	109.5
C7—C8—H8	119.7	H14B—C14—H14C	109.5
C9—C8—H8	119.7	O1—C15—C2	123.51 (18)
N1—C9—C8	118.78 (13)	O1—C15—H15	118.2
N1—C9—C4	122.64 (14)	C2—C15—H15	118.2
C8—C9—C4	118.56 (15)		
