# Supplementary figures and images for: Crystal structure of 2-(4-methyl­piperazin-1-yl)quinoline-3-carbaldehyde
Source: Acta Crystallogr E Crystallogr Commun. 2015 Oct 31;71(Pt 11):o900–1. doi: 10.1107/S2056989015020186 (PMC4645024; doi:10.1107/S2056989015020186)

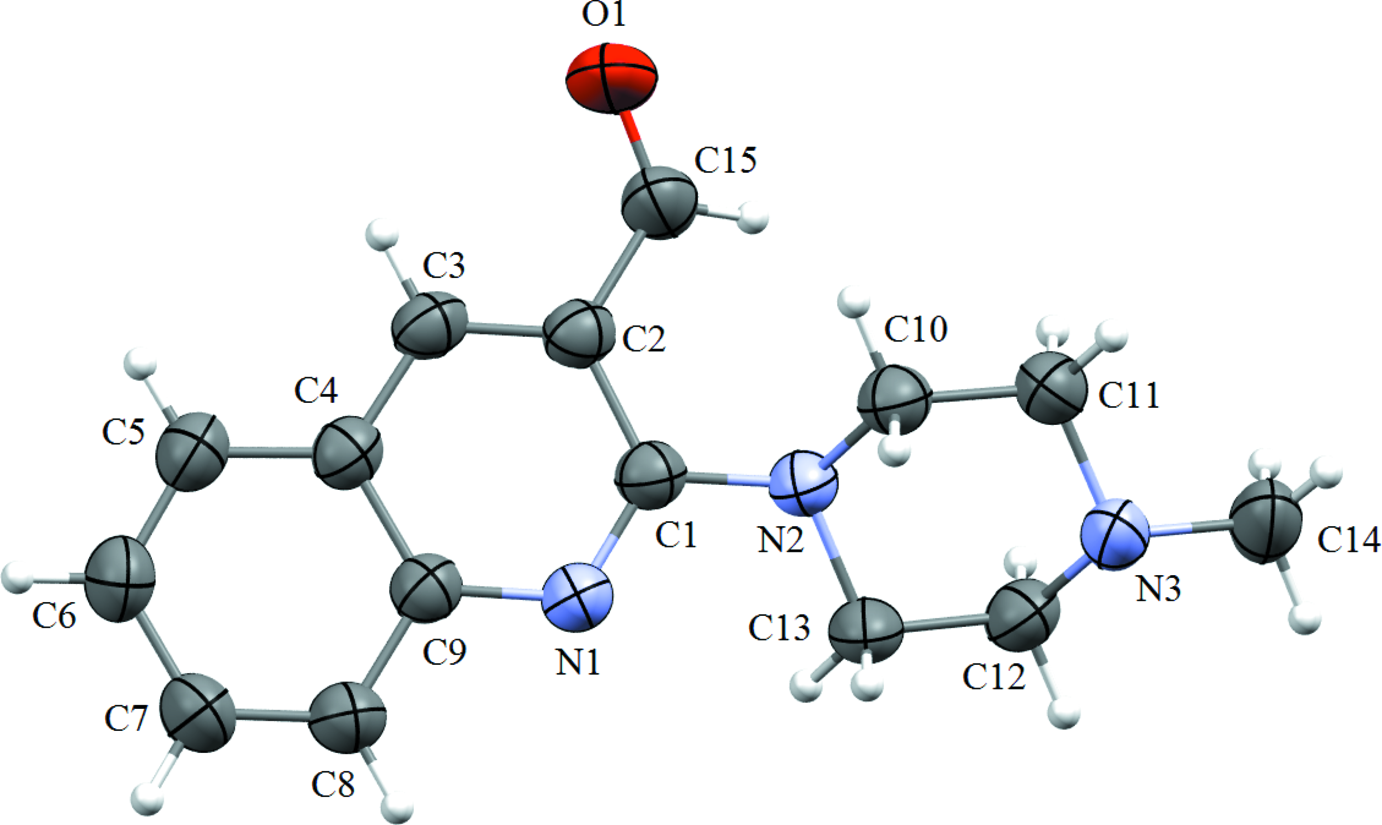

Supplement: Supplementary file 4 [file e-71-0o900-fig1.tif]
